# A New Self-Consistent Field Model of Polymer/Nanoparticle Mixture

**DOI:** 10.1038/srep20355

**Published:** 2016-02-01

**Authors:** Kang Chen, Hui-shu Li, Bo-kai Zhang, Jian Li, Wen-de Tian

**Affiliations:** 1 Center for Soft Condensed Matter Physics & Interdisciplinary Research, College of Physics, Optoelectronics and Energy, Soochow University, Suzhou 215006, China; 2 National Laboratory of Solid State Microstructures and Department of Physics, Nanjing University, Nanjing 210093, China; 3 Department of Physics, Nanjing Normal University, Nanjing 210023, China; 4 Kavli Institute for Theoretical Physics China, CAS, Beijing 100190, China

## Abstract

Field-theoretical method is efficient in predicting assembling structures of polymeric systems. However, it’s challenging to generalize this method to study the polymer/nanoparticle mixture due to its multi-scale nature. Here, we develop a new field-based model which unifies the nanoparticle description with the polymer field within the self-consistent field theory. Instead of being “ensemble-averaged” continuous distribution, the particle density in the final morphology can represent individual particles located at preferred positions. The discreteness of particle density allows our model to properly address the polymer-particle interface and the excluded-volume interaction. We use this model to study the simplest system of nanoparticles immersed in the dense homopolymer solution. The flexibility of tuning the interfacial details allows our model to capture the rich phenomena such as bridging aggregation and depletion attraction. Insights are obtained on the enthalpic and/or entropic origin of the structural variation due to the competition between depletion and interfacial interaction. This approach is readily extendable to the study of more complex polymer-based nanocomposites or biology-related systems, such as dendrimer/drug encapsulation and membrane/particle assembly.

Addition of nanofillers to polymer materials has long been a practical approach to fabricate flexible composites with enhanced mechanical, electrical or optical properties^1^,^2^,^3^,^4^,^5^,^6^. Understanding and controlling the formation of the assembling structures of polymer nanocomposites (PNCs) are the keys to realizing the desired macroscopic performance. Many theoretical and simulation methods have been developed^7^,^8^,^9^,^10^,^11^,^12^,^13^,^14^,^15^,^16^,^17^,^18^,^19^,^20^,^21^,^22^,^23^,^24^,^25^,^26^,^27^,^28^,^29^,^30^,^31^,^32^,^33^ to study the assembling behaviors of PNCs. Therein, the field-based model for PNCs^18^,^22^,^29^,^30^ is pursued due to the power of the self-consistent field theory (SCFT) in predicting the mesoscopic structures of multicomponent polymeric systems^34^,^35^,^36^. The challenges facing the incorporation of particles into the field-based model of polymers are how to treat the polymer-particle interface and how to address the strong excluded-volume (EV) interactions both between polymer and particle and between particles. The hybrid theory proposed by Thompson and coworkers couples the SCFT for polymers with the density functional theory for particles. This hybrid method has been quite successful in predicting the ordered structures of diblock copolymer/nanoparticle composites^18^,^30^,^37^,^38^,^39^. However, it’s well understood that the interfacial and EV interactions between polymer and particle are not properly considered in this approach. Another type of hybrid method treats the particles as cavities in space and combines the field description of polymers and Brownian dynamics (BD) simulation of particles^22^,^40^,^41^. This cavity model keeps the explicit particle coordinates and hence can potentially overcome the challenges mentioned above. However, the difficulty lies in the coupling between the SCFT iterations and the BD motions, i.e. how to determine the force on the particles exerted by surrounding polymers. Sides and coworkers^22^ used the *explicit partial derivative* of the Hamiltonian to the particle positions as the driving force on the particles. This is questionable because the auxiliary fields (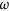
’s) in SCFT are implicit functions of the particle positions. Total derivative of the Hamiltonian to the particle positions is more appropriate in the sense of quasi-static approximation. However, the expression for this total derivative is not available. Another issue with the SCFT/BD method is that the alternating SCFT iterations and small-step BD motions make it computationally expensive. To avoid the complication of considering the EV interactions in the polymer/multi-nanoparticle system, some works focused on the dilute limit (one or two fixed particles)^28^,^42^ or made a simplification by invoking the concept of effective polymer concentration^43^.

Here, we develop a field-based model of PNCs which unifies the particle description with the polymer field. Unlike conventional field models^18^,^29^,^30^ in which particles are described by continuous density distribution, the particle density in our model is eventually discrete and can represent individual particles as in the cavity model and other particle-based methods. This kind of particle density is no longer an “ensemble-averaged” distribution but of instantaneous nature. The morphology we predict reflects the ensemble-averaged density distribution of polymers with individual nanoparticles located at the “*most probable*” positions. The motivation is three-fold: 1) patterns with instantaneous particle locations can better exhibit delicate structures (especially when the particles are packed) and be compared with experimental results; 2) recovering the particle coordinates allows proper treatment on the EV interactions and the polymer-particle interface; 3) due to 2), the evaluation of the polymer-particle interfacial energy is more appropriate and the contribution of the depletion effect can also be involved. We use this model to study the simplest system of nanoparticles immersed in dense homopolymer solution. We focus on the structural variations under diverse polymer-particle interfacial interactions and depletion thickness.

## Model and Method

We consider a system consisting of a mixture of 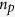 homopolymers and 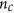 spherical solid particles. We use the grand canonical ensemble to describe the polymers, i.e. 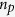 is determined by the chemical potential and may vary under different situations. While 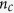 is assigned and fixed in the calculation. The radius of all particles is 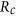. The coarse-grained polymer chain is composed of N segments of size 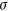. The bonded interactions of Gaussian chain are quantified by an elastic potential energy:


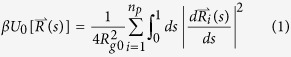


where 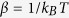, 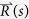 represents the configuration of the polymer chain, 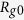 is the unperturbed radius of gyration. We employ the quadratic compressible model to address the EV interaction between segments in the presence of particles:


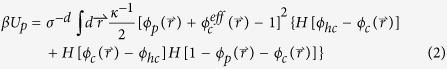


*d* is the dimension, 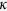 is a dimensionless parameter proportional to the compressibility of the polymer matrix. 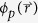 and 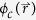 are dimensionless concentrations (local volume fractions) of segments and particles, respectively.


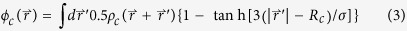


where 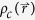 is the number density of particles. The surface profile of particles is smoothed by the hyperbolic tangent function for numerical efficiency. Note that, for conciseness, we directly use the “ensemble-averaged” symbols in the above and following energy expressions, since they are interchangeable with instantaneous ones in the mean field approximation.

**Figure 1:**
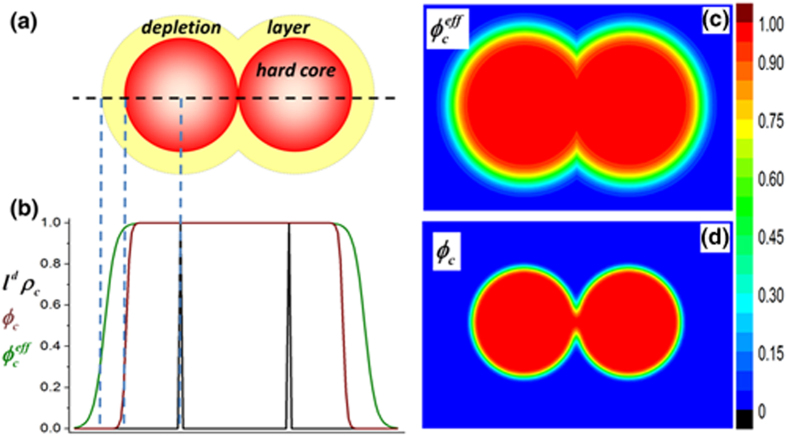
Schematic and concentration plots of two complete particles in contact in two dimensions. (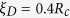). (**a**) Schematic of the two particles shows the hardcore regions and depletion layers. (**b**) Concentration profiles of 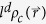 (black), 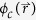 (red) and 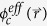 (green) along the center-to-center line of the two particles. (**c**) Concentration plot of 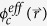. (**d**) Concentration plot of 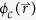.

In the calculation, the particle density gradually evolves into individual particles. And in this process it’s important to decide whether a location is in the hardcore or non-hardcore regions of particles. Hence, we introduce 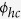 in Eq. (2) as a threshold quantity. We set 
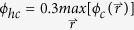
, i.e., about one third of the maximum particle concentration in the system. In the hardcore (non-hardcore) region, 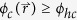 (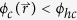). 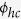 varies during the calculation and eventually, 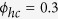. The coefficient 0.3 is empirical; other choices of the number only weakly influence the results. The Heaviside functions in the curly brackets of Eq. (2) only serve as a judgment to pick the non-hardcore regions or the hardcore regions where the total concentration is less than one. In these two cases, only the weak polymer-polymer EV interaction characterized by 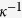 is considered. Otherwise, the strong polymer-particle EV interaction characterized by 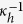 (Eq. (6)) is triggered to ensure the total concentration in the hardcore region does not exceed 1.


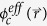
 in Eq. (2) is introduced to take into account the depletion layer surrounding the surface of each particle and the capability of the layer to overlap with each other and with the entity of particles. This capability of overlapping leads to the so-called depletion effect (see Fig. 1). We approximate that the density profile of polymers in the depletion layer is described by a hyperbolic tangent function in the case of neutral polymer-particle interfacial interaction. Equivalently, we set an effective particle concentration (not real) to mimic the depletion layer felt by polymers. The particle at position 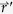 generates an effective particle concentration at position 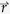:


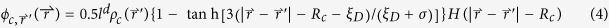


where 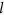 is the grid size of the simulation box. The thickness of depletion layer, 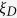, is related to the rigidity of the polymer chain^43^,^44^. The Heaviside function in Eq. (4) guarantees the position 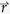 is outside the entity of the particle at position 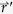. The effective particle concentration is then:


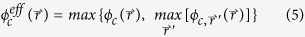


The first max function addresses the overlap between the depletion layer and the entity of particles, while the second one addresses the overlap between the depletion layers of nearby particles. Figure 1 shows the concentration plots of two contact complete particles in our model. Apparently, the particles are surrounded by a depletion layer mimicked by effective particle concentration felt by polymers. And, the depletion layers can overlap (not summation) with each other and with the entity of particles.

The strong polymer-particle EV interaction is triggered where the total concentration in the hardcore region of particles is larger than one (the first term of Eq. (6)):


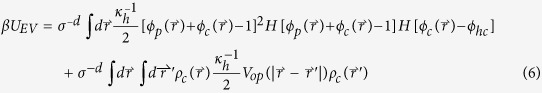


The second term of Eq. (6) represents particle-particle hardcore repulsion which is set to be proportional to the overlap “volume”, 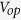, between two nearby particles. 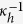 is chosen to be large enough to avoid overlap between particles and between polymer and particle. This expression for particle-particle EV repulsion is appropriate only in terms of instantaneous density, which is consistent with the instantaneous nature of the particle density in our model. Note that the ensemble-averaged particle concentrations do overlap.

The chemical nature of the polymer and particle is encoded in an interfacial energy of exponential form^25^:


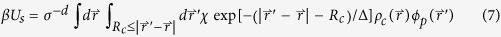



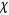
 is the dimensionless strength of the interfacial interaction or relative affinity between polymer and particle (positive for repulsion and negative for attraction). 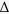 denotes the spatial range. Besides the above potentials of real origins, an artificial double-well like potential is introduced to force the formation of individual particles:


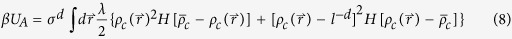



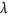
 is the strength of the two harmonic potentials which drive the particle number density at each grid in the simulation box toward 0 or 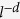. This artificial potential embodies the inseparability of a real particle. Note that this potential is position-unbiased, i.e., it does not directly influence the spatial arrangements of particles.

We fix 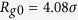. The chemical potential of polymer, 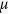, is chosen so that the concentration of the bulk polymer is 1. We set 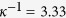, which corresponds to the compressibility of polymethylmethacrylate melt at 450 K^45^,^46^. The SCFT calculations are performed in two dimensions (see Supplementary Information for the full SCFT equations)^47^,^48^. As in the real-space screening method^48^, the calculation is started with randomly generated potential fields for both the polymer and particle. Annealing process (gradually increasing 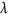) is used for the gradual formation of the individual particles and for effectively searching the morphology with low energy. Rather than predicting the configurations corresponding to the global minima, we only look for the specific morphologies showing the features of particle assembly at certain conditions.

## Results and Discussion

### Assembly of small nanoparticles

**Figure 2:**
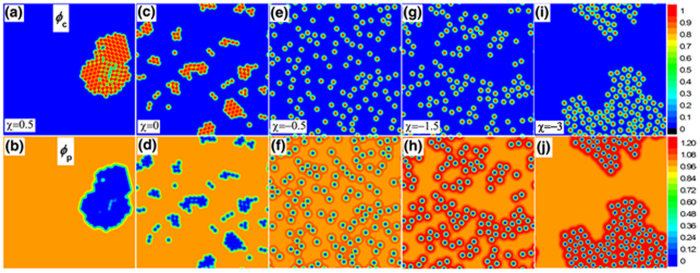
Concentration plots of nanoparticles, 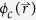 (top row) and polymers, 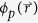 (bottom row) in two dimensions. There are 130 small nanoparticles (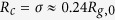) immersed in a polymer matrix with dimensions of 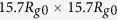 (average area fraction of particles, 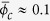). 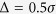 is fixed. The strength varies: 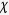
= 0.5 (**a,b**); 0 (**c,d**); −0.5 (**e,f**); −1.5 (**g,h**) and −3 (**i,j**).

We first consider the case of small nanoparticles (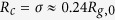). The depletion effect is neglected (by setting 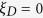), since the size of the nanoparticle is comparable to that of the segment. The typical assembly morphologies of varying the strength of interfacial interaction are shown in Fig. 2. The particle concentration in these final morphologies can represent individual particles showing clear interfaces with surrounding polymers. The packing or dispersion of these particles is directly visible. For weak repulsion, 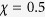 (Fig. 2(a,b)), the particles macroscopically separate from the polymer matrix and aggregate contactly (closely packed) into one large cluster (Contact Aggregation, CA) to minimize the interfacial energy. It is a typical enthalpic-driven phase separation of a binary mixture. Non-uniform clusters of contact-aggregated particles (Contact-Aggregation Clusters, CAC) are formed for very small repulsion (or 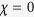, where weak entropic attraction is caused by the hyperbolic tangent surface profile of particles) (Fig. 2(c,d)). As expected^26^, in the case of weak attraction (Fig. 2(e,f)), particles are dispersed randomly in the polymer matrix, each one of which is coated with a layer of slightly more concentrated polymers (Random Dispersion, RD). As the attraction is enhanced, the bound polymer layers get denser and particles get closer to share the bound layers. This aggregation of particles is ascribed to the bridging effect^26^. Irregular domains rich in both polymers and particles (Loosely Bridging Aggregation, LBA) are formed (Fig. 2(g,h)) at intermediate attraction strength. For strong attraction (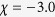), the nanoparticles aggregate closely (Closely Bridging Aggregation, CBA). The system can be viewed as composed of two separated phases: bulk polymer solution and the concentrated phase rich in both polymers and particles (Fig. 2(i,j)). Note that, intuitively, the energy of the intermediate morphologies (Fig. (c,d), (g,h)) should not be the global minima. But we still consider them as featured morphologies and ascribe their emergence to the configurational entropy of particles. This entropy takes effect during the search of the energy landscape but is missed in the energy expression.

### Morphology diagram and thermodynamic analysis

**Figure 3:**
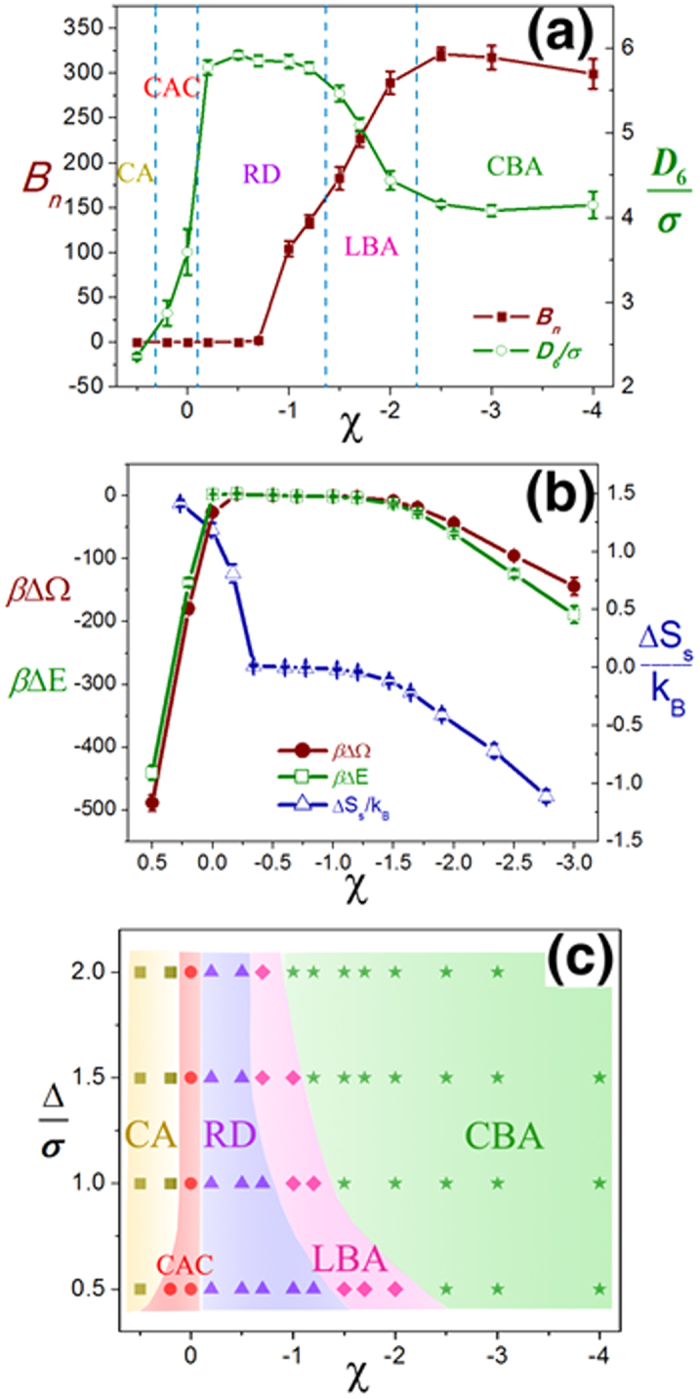
Analysis of the structural variation of small nanoparticles immersed in polymer matrix. (The system parameters are the same as in Fig. 2. Every data point is averaged over 10 independent runs.) (**a**) 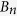 (red solid squares) and 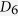 (green open circles) as functions of 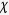 (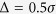). (**b**) The particle distribution of Fig. 2e is taken as the reference. The curves show the differences of grand potential (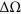, red solid circles), potential energy (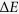, green open squares) and entropy per polymer chain (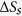, blue open triangles) between the “equilibrium” and the reference particle distributions (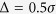). (**c**) Morphology diagram in the 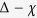 space.

To quantitatively analyze the morphologies in Fig. 2, we introduce two useful quantities: the number of pairs of bridging-connected particles, 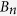 and the mean distance between a particle and its six nearest neighbors, 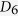. 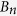 reflects the degree of bridging aggregation of particles from the view of amount. 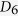 reflects the average degree of packing of particles from the view of distance. Two particles are deemed bridging-connected if a) their surface-to-surface distance is less than 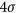, and b) the concentration of polymer in between exceeds 1.2. The choice of 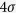 is based on the calculation of two-particle potential of mean force in the dilute particle limit, which shows the range of attraction well for 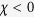 extends to 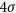. The number 1.2 (>1) is empirical, which does not influence the trend of 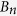. The variation of these two quantities with 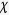 is shown in Fig. 3(a). Every data point is averaged over 10 independent runs. For different initial seeds, the overall feature of the final morphologies is the same but the packing details of particles are different, which causes a weak fluctuation in the energy and entropy (see the error bars in Fig. 3(b)). When 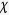 decreases from 0.5 to 0, 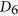 grows rapidly due to the increase of the number of particles at the interface (corresponding to the variation of the morphology from CA to CAC). A sharp transition happens between 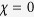 and 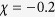 that particles are no longer in contact (i.e., the transition of the morphology from CAC to RD). For weak attraction (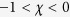), 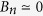 and 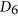 reaches a maximum plateau, which implies the well dispersion of particles in the polymer matrix. As the attraction is enhanced, 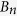 (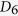) increases (decreases) gradually, indicating the formation of more and more bridging connections or the closer and closer aggregation of particles (i.e., the formation of LBA). At very large attraction, 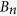 (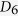) saturates at ~300 pairs of bridging connections (average center-to-center distance of ~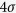), corresponding to the formation of the CBA morphology. The calculation of 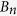 and 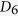 helps determine the morphology diagram in Fig. 3(c).

To thermodynamically understand the variation of the morphologies, we take the particle distribution in RD morphology, specifically the distribution of particles in Fig. 2(e), as the reference. We calculate the differences of grand potential (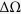), potential energy (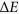) and entropy per polymer chain (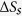) between the “equilibrium” and the reference morphology at various 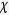 (Fig. 3(b) and see Supplementary Information for the expressions of 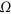, *E* and 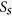). For 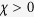, 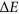 is negative and 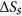 is positive, i.e., both the enthalpy and entropy are the driving force for the formation of contact aggregation of particles. The values of 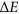 and 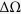 are close, which means enthalpy is the main driving force. For 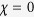, the potential energy does not change (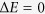) with the rearrangement of particles, and the negativity of 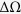 comes from the increase of entropy of polymer chains, i.e., the CAC morphology at 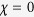 is solely entropic-driven. When 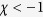, both 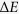 and 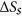 are negative, i.e., enthalpy (entropy) favors (disfavors) the bridging aggregation of particles. Figure 3(c) shows the morphology diagram in the 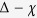 space. The regions of the intermediate morphologies, CAC and LBA, are narrow. With the increase of 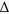, the regions of CAC, LBA and RD all shrink, manifesting that long-range interfacial interaction suppresses the dispersion of small nanoparticles in the polymer matrix.

### Depletion vs. interfacial attraction in the case of large nanoparticle

**Figure 4:**
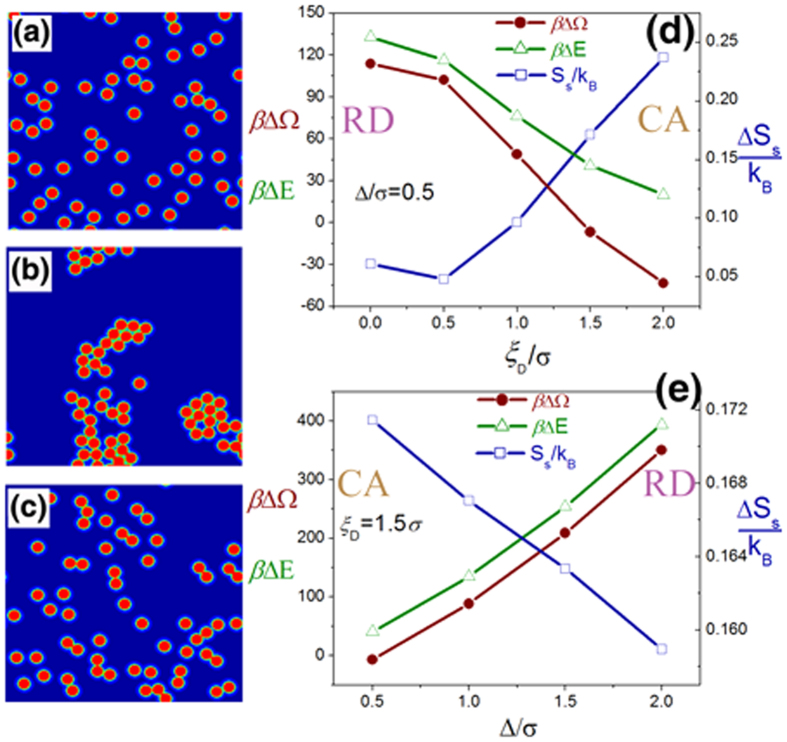
Structural variation of large nanoparticle/polymer composites in the case of weak attraction (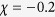). There are 60 nanoparticles (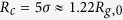) immersed in the polymer matrix with the dimensions of 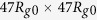 (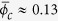). Three concentration plots of nanoparticles are for (**a**) 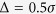, 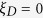; (**b**) 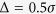, 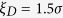; and (**c**) 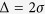, 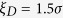. The differences of grand potential (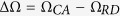, red solid circles), potential energy (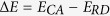, green open triangles) and entropy per polymer chain (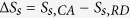, blue open squares) between the “standard” particle distributions (RD and CA) are shown along two paths: (**d**) 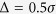 and varying 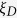; (**e**) 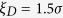 and varying 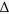.

We also investigate large nanoparticles immersed in the polymer matrix. The radius of particles is set to be 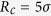 (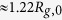). Since the particle is much larger than the segment, we turn on the depletion effect, by controlling the parameter 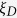. Larger 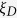 implies more rigid polymer chain^43^,^44^. Here, we focus on the competition between the depletion and the interfacial interaction. When 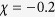, the system is close to the boundary between RD and CA phases. Particle distributions for 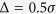 and 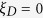, 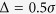 and 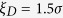, 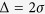 and 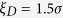 are shown in Fig. 4(a–c), respectively. The particle distribution changes from RD to CA along the path of increasing 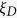 at constant 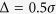. While, on the contrary, the distribution changes from CA to RD along the path of increasing 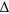 at constant 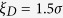. Hence, we have the conclusion that narrow depletion layer (or flexible polymer chains) and/or “long”-range weak polymer-particle attraction can facilitate the dispersion of large nanoparticles into polymer matrix.

We take the particle distributions of Fig. 4(a,b) as the “standard” RD and CA distributions, respectively and calculate the differences of 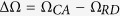, 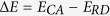 and 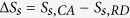 along the two paths: varying 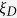 with constant 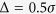 and varying 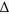 with constant 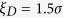 (Fig. 4(d,e)). Both 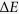 and 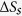 at all cases are positive, i.e., the aggregation of particles is energetically unfavored but entropically favored (depletion effect). In Fig. 4(d), the transition from RD to CA happens between 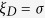 and 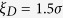 where 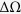 becomes zero. 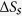 (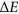) increases (decreases) with 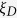, implying that the increase of depletion thickness (rigidity of polymer chain) enhances the aggregation of particles not only through the well-known depletion entropic effect but also through the alleviation of the penalty of interfacial energy. The transition from CA to RD in Fig. 4(e) happens at 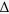 slightly larger than 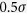. The decrease of 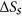 and the increase of 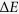 imply that the contribution of enthalpy (entropy) in the structural variation is enhanced (weakened) with the increase of 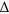.

## Conclusion

We have introduced a field model of PNCs which realizes the discrete description of particles in the final morphology. It is able to predict the collective assembly of particles in the polymer matrix with careful consideration of the EV interactions, depletion effect and interfacial interaction. This model allows investigations of the bridging and depletion effects on the multi-particle level instead of calculations based on two-particle correlations^25^,^26^. It is also able to reveal the entropic and enthalpic contributions in the variation of morphologies. Overall, it is a valuable approach to explore and analyze the rich mesostructures or particle-induced defects^49^ in polymer-based nanocomposites and is also readily extendable to the study of biology-related systems, such as dendrimer/drug encapsulation^50^ and membrane/particle assembly^51^.

## Additional Information

**How to cite this article**: Chen, K. *et al.* A New Self-Consistent Field Model of Polymer/Nanoparticle Mixture. *Sci. Rep.* **6**, 20355; doi: 10.1038/srep20355 (2016).

## Supplementary Material

Supplementary Information
