# Supplementary material for: A New Self-Consistent Field Model of Polymer/Nanoparticle Mixture
Source: Sci Rep. 2016 Feb 1;6:20355. doi: 10.1038/srep20355 (PMC4734332; doi:10.1038/srep20355)
Supplement: Supplementary Information [file srep20355-s1.pdf]

Supplementary Information

A New Self-Consistent Field Model of Polymer/Nanoparticle Mixture

Kang Chen[1,4,*], Hui-shu Li[1], Bo-kai Zhang[2,1], Jian Li[3,1] and Wen-de Tian[1,4,*]

[1]Center for Soft Condensed Matter Physics & Interdisciplinary Research, College of Physics, Optoelectronics and Energy, Soochow University, Suzhou 215006, China.

[2]National Laboratory of Solid State Microstructures and Department of Physics, Nanjing University, Nanjing 210093, China

[3]Department of Physics, Nanjing Normal University, Nanjing 210023, China

[4]Kavli Institute for Theoretical Physics China, CAS, Beijing 100190, China

[*]Authors to whom Correspondence should be addressed. Electronic mail: kangchen@suda.edu.cn (K.C.); tianwende@suda.edu.cn (W.d.T.)

In our model, the system of simple nanoparticle/polymer mixture is specified by nine parameters: σ (segment size), $R_{g,0}$ (unperturbed radius of gyration of polymer chain), R_c (radius of particles), ξ_D (thickness of the depletion layer), Δ (spatial range of polymer-particle interfacial interaction), n_c (number of particles), μ (chemical potential of polymers), κ^{-1} (dimensionless parameter proportional to the compressibility of the polymer matrix) and χ (strength of polymer-particle interfacial interaction). We set σ as the unit length and fix $R_{g,0}=4.08$ (N=100). μ is chosen that the volume fraction of bulk polymers is 1. κ^{-1} varies with, such as, the concentration of the polymer solution or the temperature. Its value influences the results at a quantitative level. We set $\kappa^{-1}=3.33$ which corresponds to the compressibility of polymethylmethacrylate (PMMA) melt at 450K.

SCFT is a very powerful approach in predicting the mesoscopic structures of multicomponent polymeric systems. In our model, we employ the grand-canonical description for the polymer solution and canonical description for the nanoparticles. Given the interaction potentials [Eqs.(1), (2) and (6-8) in the manuscript], we follow the SCFT approach and obtain the mean-field grand potential:

$$\begin{aligned}\beta\Omega_0 = \beta U_p + \beta U_c + \beta U_s + \beta U_A - \sigma^{-d}\int d\vec{r} w_p(\vec{r})\phi_p(\vec{r}) - \int d\vec{r} w_c(\vec{r})\rho_c(\vec{r}) \\ - z_p Q_p\left[w_p(\vec{r})\right] - n_c \ln\left\{Q_c\left[w_c(\vec{r})\right]/n_c\right\}\end{aligned} \tag{S1}$$

$w_p(\vec{r})$ and $w_c(\vec{r})$ are auxiliary fields to decouple interactions; $z_p = e^{\beta\mu}$ is the activity of polymers; Q_p and Q_c are the partition functions of a single polymer chain or nanoparticle in the auxiliary fields, respectively. The fields are given by

$$\begin{aligned}w_p(\vec{r}) = \kappa^{-1}\left[\phi_p(\vec{r}) + \phi_c^{eff}(\vec{r}) - 1\right]H_0(\vec{r}) + \kappa_h^{-1}\left[\phi_p(\vec{r}) + \phi_c(\vec{r}) - 1\right]H_h(\vec{r}) \\ + \int_{R_c \le |\vec{r}-\vec{r}'|} d\vec{r}'\varepsilon \exp\left[-\left(|\vec{r}-\vec{r}'| - R_c\right)/\Delta\right]\rho_c(\vec{r}')\end{aligned} \tag{S2}$$

$$
\begin{aligned}
w_c(\vec{r}) = &\sum_{\vec{r}'} \sigma^{-d} l^d \kappa^{-1} \left[\phi_p(\vec{r}') + \phi_c^{eff}(\vec{r}') - 1\right] Th(\xi_D) H\left(\left|\vec{r}-\vec{r}'\right| - R_c\right) \delta\left[\phi_{c,\vec{r}}(\vec{r}'), \phi_c^{eff}(\vec{r}')\right] H_0(\vec{r}') \\
&+ \sigma^{-d} \int d\vec{r}' \kappa^{-1} \left[\phi_p(\vec{r}') + \phi_c^{eff}(\vec{r}') - 1\right] Th(0) \delta\left[\phi_c(\vec{r}'), \phi_c^{eff}(\vec{r}')\right] H_0(\vec{r}') \\
&+ \sigma^{-d} \int d\vec{r}' \kappa_h^{-1} \left\{\left[\phi_p(\vec{r}') + \phi_c(\vec{r}') - 1\right] Th(0) H_h(\vec{r}') + V_{op}\left(\left|\vec{r}-\vec{r}'\right|\right) \rho_c(\vec{r}')\right\} \\
&+ \sigma^{-d} \int_{R_c \le \left|\vec{r}'-\vec{r}\right|} d\vec{r}' \, \varepsilon \exp\left[-\left(\left|\vec{r}'-\vec{r}\right| - R_c\right)/\Delta\right] \phi_p(\vec{r}') \\
&+ \sigma^{-d} \lambda \left\{\left[\rho_c(\vec{r}) - \rho_l\right] H\left[\bar{\rho}_c - \rho_c(\vec{r})\right] + \left[\rho_c(\vec{r}) - \rho_u\right] H\left[\rho_c(\vec{r}) - \bar{\rho}_c\right]\right\}
\end{aligned}
$$

(S3)

where

$$H_0(\vec{r}) \equiv H\left[\phi_{hc} - \phi_c(\vec{r})\right] + H\left[\phi_c(\vec{r}) - \phi_{hc}\right] H\left[1 - \phi_p(\vec{r}) - \phi_c(\vec{r})\right] \tag{S4}$$

$$H_h(\vec{r}) \equiv H\left[\phi_p(\vec{r}) + \phi_c(\vec{r}) - 1\right] H\left[\phi_c(\vec{r}) - \phi_{hc}\right] \tag{S5}$$

$$Th(x) \equiv 0.5\left\{1 - \tanh\left[3\left(\left|\vec{r}-\vec{r}'\right| - R_c - x\right)/(x+\sigma)\right]\right\} \tag{S6}$$

The function $\delta[...]$ in Eq. (S3) is the Kronecker delta function. The distributions of polymers and particles are given by

$$\phi_p(\vec{r}) = \frac{z_p N \sigma^d}{V} \int_0^1 ds q(\vec{r}, s) q(\vec{r}, 1-s) \tag{S7}$$

$$\rho_c(\vec{r}) = \frac{n_c}{V Q_c} \exp\left[-w_c(\vec{r})\right] \tag{S8}$$

The chain propagator $q(\vec{r}, s)$ satisfies the diffusion equation:

$$\frac{\partial q(\vec{r}, s)}{\partial s} = R_{g,0}^2 \nabla^2 q(\vec{r}, s) - N w_p(\vec{r}) q(\vec{r}, s) \tag{S9}$$

with the initial condition $q(\vec{r}, 0) = 1$. The partition functions of a single polymer chain and nanoparticle are given by

$$Q_p\left[w_p(\vec{r})\right] = \frac{1}{V} \int d\vec{r} q(\vec{r}, 1) \tag{S10}$$

$$Q_c\left[w_c(\vec{r})\right] = \frac{1}{V} \int d\vec{r} \exp\left[-w_c(\vec{r})\right] \tag{S11}$$

The Eqs. (S1)-(S11) consist the set of SCFT equations. To find the numerical solutions, we first make a random initialization for the auxiliary fields and then update the densities or concentrations and auxiliary fields iteratively.[47] Pseudo spectral method is adopted to solve the diffusion equation.[46] This iterative procedure is coupled with an "annealing" process (varying λ in Eq.(8) in the manuscript) for the artificial potential, which controls the gradual formation of discrete particles. In the final morphology, the particle density is of instantaneous nature and can represent individual particles. Therefore, we remove the particle-entropy contribution in the grand potential, i.e.,

$$\beta\Omega = \beta U_p + \beta U_c + \beta U_s + \beta U_A - \sigma^{-d} \int d\vec{r} w_p(\vec{r}) \phi_p(\vec{r}) - z_p Q_p \left[w_p(\vec{r}) \right] \quad \text{(S12)}$$

The grand potential of Eq. (S12) is the grand potential we calculate in this paper. The single-chain entropy is defined as $S_s / k_B = \ln(Q_p) - 1/(\sigma^d n_p) \int d\vec{r} w_p(\vec{r}) \phi_p(\vec{r})$.
